# Validation of SNP Allele Frequencies Determined by Pooled Next-Generation Sequencing in Natural Populations of a Non-Model Plant Species

**DOI:** 10.1371/journal.pone.0080422

**Published:** 2013-11-07

**Authors:** Christian Rellstab, Stefan Zoller, Andrew Tedder, Felix Gugerli, Martin C. Fischer

**Affiliations:** 1 Biodiversity and Conservation Biology, Swiss Federal Research Institute WSL, Birmensdorf, Switzerland; 2 Genetic Diversity Centre, ETH Zürich, Zürich, Switzerland; 3 Institute of Evolutionary Biology and Environmental Studies and Institute of Plant Biology, University of Zürich, Zürich, Switzerland; 4 Institute of Integrative Biology, ETH Zürich, Zürich, Switzerland; CNRS/University Lyon 1, France

## Abstract

Sequencing of pooled samples (Pool-Seq) using next-generation sequencing technologies has become increasingly popular, because it represents a rapid and cost-effective method to determine allele frequencies for single nucleotide polymorphisms (SNPs) in population pools. Validation of allele frequencies determined by Pool-Seq has been attempted using an individual genotyping approach, but these studies tend to use samples from existing model organism databases or DNA stores, and do not validate a realistic setup for sampling natural populations. Here we used pyrosequencing to validate allele frequencies determined by Pool-Seq in three natural populations of *Arabidopsis halleri* (Brassicaceae). The allele frequency estimates of the pooled population samples (consisting of 20 individual plant DNA samples) were determined after mapping Illumina reads to (i) the publicly available, high-quality reference genome of a closely related species (*Arabidopsis thaliana*) and (ii) our own *de novo* draft genome assembly of *A. halleri*. We then pyrosequenced nine selected SNPs using the same individuals from each population, resulting in a total of 540 samples. Our results show a highly significant and accurate relationship between pooled and individually determined allele frequencies, irrespective of the reference genome used. Allele frequencies differed on average by less than 4%. There was no tendency that either the Pool-Seq or the individual-based approach resulted in higher or lower estimates of allele frequencies. Moreover, the rather high coverage in the mapping to the two reference genomes, ranging from 55 to 284x, had no significant effect on the accuracy of the Pool-Seq. A resampling analysis showed that only very low coverage values (below 10-20x) would substantially reduce the precision of the method. We therefore conclude that a pooled re-sequencing approach is well suited for analyses of genetic variation in natural populations.

## Introduction

During the past few years, the use of next-generation sequencing technologies (NGS, [1]) has dramatically increased. At the same time, costs per sequenced base pair (bp) are rapidly decreasing, which facilitates genome-wide analyses of genetic variation of individuals and entire populations (e.g., [2,3]). NGS allows studies to expand to a truly genome-wide scale, potentially analyzing millions of polymorphisms at a time (e.g., [2,4,5]). However, studies at the population level typically require the analysis of numerous individuals of multiple populations, which still leads to high costs, high DNA requirements, long processing times due to library preparation, and the need for high sequence coverage per individual. Therefore, a pooled approach using samples consisting of several individuals might be preferable in many study designs.

Recently, Futschik and Schlötterer [6] demonstrated that sequencing of pooled samples (Pool-Seq) using NGS not only reduces costs and workload, but can also reliably detect single nucleotide polymorphisms (SNPs) and accurately estimate various population genomic parameters. Several studies have used Pool-Seq to answer challenging questions in evolutionary ecology (e.g., [4,5,7,8]) and adapted existing analytical concepts to this new approach [9-13]. However, the benefits of Pool-Seq come with some trade-offs. Most obviously and importantly, it leads to the loss of information regarding individual haplotypes and heterozygosity. Secondly, rare alleles that are important in medical sciences for example, might be ignored as a consequence of stringent corrections for sequencing errors (for critical comments, see 14). Thirdly, Pool-Seq requires that individual DNA samples are combined in equimolar concentrations, and therefore measurement and/or pipetting errors might have a negative impact on allele frequency estimates, at least in small pools [6]. 

Hence, studies using Pool-Seq imply that their SNP allele frequency estimates are accurate, often without validation of these allele frequency estimates. Several studies have tried to address this topic in the recent past and validated allele frequencies from pooled samples by genotyping single individuals from within the pools (see Table 1 for an overview). However, several studies used samples from existing model organism databases and DNA stores [15-18], for which polymorphisms were known in advance. Some studies tested other aspects or different variants of the NGS sequencing protocol [15,18,19], and most of the studies pre-amplified a certain region in the genome or reduced the size of the template prior to sequencing [16,17,19-24]. To our knowledge, except for Zavodna et al. [24] who looked at population genetic parameters in two mitochondrial genes, no study has been published to date that explicitly validates pooled SNP allele frequencies in natural populations of a non-model species (defined here as species without a publicly available reference genome). However, precisely this information is needed for currently hot topics in evolutionary biology, like population genomic studies of local adaptation.

**Table 1:** Overview of Pool-Seq validation studies.

**Study**	**Year**	**Study species**	**Pool-Seq technology**	**Validation by**	**Number of loci**	**Pool size**	**Result^a^**	**Remarks**
Van Tassell et al. [19]	2008	*Bos taurus*	Illumina CSMA	Illumina Infinium Array	23'357 SNPs	15-35	*r* = 0.67	Reduced representation libraries
Holt et al. [45]	2009	*Salmonella*	Illumina GA I	Illumina GA I	403 SNPs	6	*R* ^2^ = 0.92-0.95	
Druley et al. [16]	2009	*Homo sapiens*	Illumina GA I	TaqMan/database (samples with previously known polymorphisms, determined by Sanger)	14 SNPs in 4 genes	1’111	*R* ^2^ = 0.96	Pre-amplification of loci
Ingman and Gyllensten [17]	2009	*Homo sapiens*	Roche 454 GS FLX	Database	16 SNPs in 1 gene	96	*R* ^2^ = 0.88-0.91^b^	Pre-amplification of gene, tested pooled DNA and PCR products
Out et al. [22]	2009	*Homo sapiens*	Illumina GA I	Sanger	23 SNPs in 1 gene	287	*r* = 0.86^c^	Tested pooled DNA and PCR products
					17 SNPs in 1 gene^b^	88^b^	*r* = 0.99^b^	
Bansal et al. [15]	2011	*Homo sapiens*	Illumina GA IIx	HapMap database (samples with previously known polymorphisms)	>4’000 SNPs	20	*R* ^2^ > 0.99	In-solution hybridization
Margraf et al. [20]	2011	*Homo sapiens*	Illumina GA IIx	Sanger	47 SNPs in 1 gene	30/50	All known variants detected	Pre-amplification of two gene regions
Niranjan et al. [21]	2011	*Homo sapiens*	Illumina GA IIx	Sanger	n.a.	20/40	*mcc* = 0.78	Pre-amplification of gene
Zhu et al. [18]	2012	*Drosophila melanogaster*	Illumina GA IIx	Database (strains with previously known polymorphisms)	100*1’000 random SNPs genome-wide	22-92	*cc* = 0.70-0.87	Pooled flies prior to DNA extraction
Gautier et al. [23]	2013	*Thaumetopoea pityocampa*	Illumina HiSeq 2000	Illumina HiSeq 2000	49’597 SNPs	20/30	*r* = 0.93-0.99	Restriction site-associated DNA (RAD) sequencing
Zavodna et al. [24]	2013	*Leiopelma hochstetteri*	Roche 454 GS FLX	Sanger	2 mitochondrial genes	2-13	R^2^ = 0.31-0.96^d^	Pre-amplification of genes

a *R* ^2^ = determination coefficient of a linear regression, *r* = Pearson's correlation coefficient, *mcc* = Matthew's correlation coefficient, cc = concordance correlation.

b for pooled PCR products.

c calculated from the supporting material.

d comparing estimates of nucleotide diversity and pairwise population differentiation.

In the present study, we filled this gap and used pyrosequencing [25,26] of individual samples to test the accuracy of estimates of SNP allele frequencies determined by Illumina Pool-Seq. The pyrosequencing method is based on the same technological principle as Roche 454 sequencing, but uses sequencing primers specific to a particular genomic region previously amplified in a PCR reaction. Pyrosequencing has been shown to be a powerful tool for analyzing SNPs in individuals [27] or in artificially [28] and naturally pooled samples [29]. Using 20 individuals in each of three populations of the non-model species *Arabidopsis halleri* (Brassicaceae), we compared the allele frequencies at nine SNPs located within six different genes using Illumina Pool-Seq data with the allele frequencies from individual genotyping using pyrosequencing of the same 20 samples per population (in total 540 samples). The allele frequencies from the Pool-Seq were determined using two different mapping approaches. We first mapped reads to a closely related species with a high-quality reference genome (*Arabidopsis thaliana*), and second to our own *de novo* draft assembly of the *A. halleri* genome. Our results show that estimates of allele frequencies measured in the pooled samples are highly accurate, independent of the reference genome used for mapping, and that a Pool-Seq approach is therefore well suited for population genomic analyses of natural populations. 

## Materials and Methods

### Study system

*Arabidopsis halleri* (L.) is a member of the Brassicaceae and closely related to the model species *A. thaliana*. It occurs in a wide range of habitats, including mountain slopes, forest margins and rocky crevices [30]. The perennial, insect-pollinated and strictly outcrossing herb is distributed from Europe to eastern Asia [31] and has been used in studies on evolutionary ecology [32] and heavy metal tolerance [33,34] due to its ability to hyperaccumulate Zinc and Cadmium. 

We sampled 20 individuals in each of three natural populations in the southern Swiss Alps: in Brusio (46.27767°N/10.10619°E, 1070 m a.s.l.), Chironico (46.41593°N/ 8.82605°E, 850 m a.s.l.), and Castasegna (46.33682°N/ 9.52171°E, 790 m a.s.l.). A sampling permission for these locations was not required, because they do not represent nature reserves and *A. halleri* is not protected or endangered in Switzerland. Genomic DNA was extracted from dried leaves using the DNeasy Plant Kit (Qiagen). The quality of the extractions was tested using 1.5% agarose gels stained with GelRed (Biotum) and a Nanodrop 8000 (Thermo Scientific). The precise double stranded DNA concentration of individual samples was quantified using a Qubit fluorometer (dsDNA BR, Invitrogen).

### Illumina Pool-Seq

Per population, one pooled sample with a total of 7 µg of RNA-free genomic high-quality DNA was prepared with equimolar concentrations of all 20 individual samples. Library preparations (250–300 bp insertion size; 100 bp paired-end reads) and genome sequencing (HiSeq2000, Illumina) were performed by GATC Biotech (Constance, Germany). To correct for channel bias, the three tagged populations were distributed among two full channels on one flow cell. After sequencing, forward and reverse reads were trimmed with Cutadapt [35], removing tags and adaptors. Moreover, we performed quality trimming with the FASTX-toolkit (http://hannonlab.cshl.edu/fastx_toolkit), keeping only reads with high-confidence bases (based on Phred-type quality scores Q20) and no adaptors. Finally, we re-synchronized the forward and reverse read files with an in-house perl script. 

### Allele frequencies from Pool-Seq reads mapped to a closely related genome

The paired-end reads of each population were mapped to the *A. thaliana* reference genome (TAIR10, [36,37]), using BWA aln and sampe [38], allowing for 10% mismatch, and excluding organellar DNA. Only unambiguously mapped reads were kept. We then produced a multiple pileup file with SAMtools v0.1.18 (mpileup, [39]), synchronizing and filtering for base quality (Q20) with the perl script mpileup2sync.pl of PoPoolation2 [9], in order to call the SNPs. The minimum minor allele count was set to four to account for sequencing errors. Additionally, a minimum coverage of 20 and a maximum coverage of 400 per population were used as thresholds for SNP identification. This was done to accurately estimate allele frequencies and correct for potential errors caused by repeated sequences. SNP allele frequencies along the assembly were determined with snp-frequency-diff.pl in PoPoolation2.

### Allele frequencies from Pool-Seq reads mapped to the *A. halleri* de novo draft assembly

We assembled our own draft genome of *A. halleri* from the paired-end reads of one population pool (Brusio) using the software Velvet [40]. We tested various k-mers to identify the best parameters for the *A. halleri* genome assembly, which was then used as a new reference to re-map the reads from the three study populations as described above, with one exception, namely that the mismatch parameter was lowered to 5%. SNPs were called as described above. The gene sequences of the six genes of interest (Table 2, see below for more information) were downloaded from the TAIR10 database [37]. We then identified the contigs containing these genes by a local nucleotide and protein BLAST (version 2.2.26+) search. The actual SNPs of interest were located by a pairwise alignment of the contigs of interest with the “sequence to analyse” used for pyrosequencing (Table 2) with the software Geneious 6.1.6 (Biomatters Ltd.; www.geneious.com).

**Table 2:** SNP positions, PCR and pyrosequencing primers.

Gene	TAIR locus identifier	SNP	SNP position in consensus sequence^a^ [bp]	Contig name^b^	SNP position in contig^c^ [bp]	Primer	Primer sequence 5’-3’	PCR product size [bp]	T_A_^d^[°C]	Sequence to analyse^e^	Pyrosequencing failures (of 60 samples)
ADC2	AT4G34710	1	1587	NODE_171306	9158	PCR forward	CGGCGACACCATCAGAGA	223	58		
						PCR reverse	*bio-GTGAGGCCTGTCATTGGG*				
						Pyrosequencing	ACAATCTGAGTAGTAGTCAA			**W**CCAAATTTGCCCTT	0
AHK1	AT2G17820	1	1251	NODE_107322	14799	PCR forward	*bio-*ATGACATGCCCTTTTCGTCTAAGT	163	58		
						PCR reverse	TCATAGTGGTGGATATCGGAGTAC				
						Pyrosequencing	AACTACCTCGACAGATTCT			CCA**W**CAAAGC AAA	0
AHK1	AT2G17820	2	4933	NODE_107322	18437	PCR forward	*bio-CCTTGTGAAATGCCATGTTAGAA*	373	55		
						PCR reverse	ATGGGTTACCCCTTGGAGAAGT				
						Pyrosequencing	TGGAGAGTCCAAGAGAG			ATGATGAT**W**TTGC	2
AT3G60750	AT3G60750	1	1059	NODE_134451	1382	PCR forward	*bio-*GAAGCAAGACACCAGGACATC	197	58		
						PCR reverse	CCAACAGCATTAGCGATTCCT				
						Pyrosequencing	GAAATTTTACATGAACCATA			**R**GCCAAAAGAAGC	0
ERD7	AT2G17840	1	1622	NODE_8629	4361	PCR forward	CTTGACCTTTGGAAGCAATTGTA	106	58		
						PCR reverse	*bio-ACCAAAGAAATCGCACATGA*				
						Pyrosequencing	AATTGTAAGTCCGTAGTTCA			A**Y**ATCTCGTTAGTGTTTTT	8
RUS1	AT3G45890	1 & 2	1982/1996	NODE_69242	24280/24294	PCR forward	GAACGGCTTCAACTAGGGTCA	129	58		
						PCR reverse	*bio-GCTTACGCAGAATCTTCCTCTGT*				
						Pyrosequencing	AACAAGGAAGAAGCAATAG			CA**Y**TGTTTGATCTGTA**Y**CGC	6/5
RUS1	AT3G45890	3	2430	NODE_69242	24722	PCR forward	TACCAACCAGAATCCGTCTAGG	96	58		
						PCR reverse	*bio-ATCATCCATTGGCCCTGGT*				
						Pyrosequencing	CAGCTCATAAACTCAGAG			**S**AAAACCAGGGCCAA	0
UHV1	AT5G41150	1	3583	NODE_76279	4123	PCR forward	CCTGAGACACATCCAACTGAAAC	99	55		
						PCR reverse	*bio-CTATGGTCTCCGGTTTCGC*				
						Pyrosequencing	ACTGAAACCTTGGCC			A**W**AGMTGGAGYCT	1

a Positions of the SNPs in the consensus sequences of File S1.

b Names of the contigs containing the SNPs (File S2).

c Positions of the SNPs in the contigs of File S2.

d Annealing temperature.

e The investigated SNPs are marked bold and underlined (IUPAC Codes).

### SNP genotyping from individual samples using pyrosequencing

The nine SNPs tested here were selected (using the *A. thaliana* genome as reference for the mapping) during a process of finding highly differentiated SNPs in genes (Table 2) that are presumably under habitat-mediated selection (Fischer et al., unpublished results). Based on the mapped consensus sequences provided in File S1, we developed eight pyrosequencing assays (Table 2) to cover the nine SNPs with PyroMark Assay Design 2.0 (Qiagen), including one assay that covered two closely situated SNPs in gene *RUS1*. To avoid mispriming, all potential PCR primer sequences were BLASTed against the publicly available *A. thaliana* genome as well as the *A. halleri* de novo draft assembly, checking for potential PCR fragments smaller than 3’000 bp using primer sites that have at most three mismatches. Additionally, PCR products were checked for single bands on an agarose gel.

Prior to pyrosequencing, we performed a PCR for each assay in a 40 µL reaction volume containing 1 µL DNA, 0.125 mM of each dNTP (Life Technologies), 0.2 µM of each primer (Microsynth; Table 2), 2.0 mM MgCl_2_ (Life Technologies), 1 U of AmpliTaq Gold DNA Polymerase (Life Technologies) and 1x buffer. An initial polymerase activation step of 5 min at 95°C was followed by 50 cycles of 15 s at 95°C, 30 s at the primer-specific annealing temperature (Table 2) and 20 s at 72°C, and a final step of 4 min at 72°C. The biotinylated PCR products were extracted with streptavidin sepharose beads (GE Healthcare) according to the manufacturer's instructions and released into a PSQ 96 Low Plate (Biotage) containing 39 µL annealing buffer (20 mM Tris-Acetate, 2 mM Mg-Acetate, pH = 7.6) and 1 µL of 10 µM pyrosequencing primer (Table 2). The plate was incubated at 80°C for 2 min, cooled to room temperature and run on a PyroMark ID machine (Biotage) using PyroMark Gold reagents (Qiagen) as specified by the manufacturer. The dispensation order of the four nucleotides was determined automatically by the PyroMark ID software (Biotage), and genotyping was performed using default settings. In total, we performed 480 sequencing reactions (8 assays × 60 individuals), resulting in 540 individual loci.

### Statistical analyses

The following analyses were performed in IBM SPSS Statistics 21 for both mapping approaches, i.e. using the allele frequencies derived from mapping to *A. thaliana* and to our *A. halleri* draft genome. To assess the accuracy of SNP frequency estimates based on the Pool-Seq approach, we performed a linear regression between major allele frequencies (MAF) estimated on the basis of Pool-Seq data and population allele frequencies determined from individual samples genotyped by pyrosequencing. The major allele was defined as the allele that showed the highest frequency from the reads of all three populations combined. Therefore, for single populations, MAF can be below 0.5. The regression included 27 (3 populations × 9 SNPs) comparisons. Since we were testing technical aspects, we considered all comparisons as independent detection events, although they are biologically not independent. As we were mainly interested in the deviation from the ideal 1:1 ratio of the two estimates of allele frequency, we did not transform the data when determining the intercept, slope and *R* ^2^-value of the regression. However, for the calculation of a *p*-value for the regression, we used arcsine square root-transformed allele frequencies. To check if Pool-Seq generally under- or overestimates allele frequencies, we performed a paired *t*-test on the 27 comparisons using arcsine square root-transformed allele frequencies. Both the linear regression and the paired *t*-test were performed for all 27 comparisons and for the comparisons without missing values only (amplification and/or sequencing failures in the pyrosequencing process). This allowed us to estimate the effect of missing values on the results. Finally, we tested the effect of sequencing coverage on the accuracy of the estimates of allele frequency by using a correlation analysis (Pearson's *r*) with the coverage as the explaining variable and the absolute difference between the two estimates of allele frequency as the dependent variable.

As the coverage analysis described above only included SNPs with high coverage, we used a resampling approach to test the (theoretical) effect of low coverage on the accuracy of the Pool-Seq allele frequency estimates. For this analysis, only reads that mapped to the *A. thaliana* reference genome were used. With an in-house script in R 3.0.1 [41], we randomly drew reads from each of the 27 SNP/population combinations using a coverage range of 1 to 55x and 1000 iterations. For each iteration and coverage, we determined the allele frequencies and performed a linear regression between these allele frequencies and those obtained from pyrosequencing. We report average values of the mean allele frequency difference and the *R* ^2^ of the linear regressions for each simulated coverage.

## Results

### Estimates of allele frequencies from Pool-Seq

The sequencing of the three pooled population samples resulted in over 390 million paired-end reads after adaptor and quality trimming. This corresponds to 78.0 Gb of sequence data. The median Phred-score was 37 and the overall coverage was 312x for the haploid *A. halleri* genome (average of 104x per population). We unambiguously mapped 18.1% of the *A. halleri* reads to the *A. thaliana* reference genome. Using a minimum minor allele count of four, we identified over 2 million SNPs among the three populations. The coverage at the nine SNPs selected for the validation (Table 2 and File S1) was on average 119x per population, with a range of 55 to 269x for the mapping against *A. thaliana*.

The best (partial) *de novo* assembly for *A. halleri* was obtained with a k-mer of 85. In total, 175.7 Mb were assembled in 102’508 contigs that were larger than 200 bp. The longest contig had 115’790 bp and N_50_ was 5’775 bp. All six genes and nine SNPs of interest (originally detected by mapping to the *A. thaliana* genome) could be identified in the contigs of the *de novo* assembly (Table 2 and File S2). The coverage at these SNPs was on average 120x, with a range of 69 to 284x. The size of the contigs identified by BLAST search containing these genes and SNPs ranged from 5’368 to 38’746 bp.

### SNP genotyping from individual samples using pyrosequencing

The eight pyrosequencing assays (Table 2) developed for genotyping the nine SNPs in six different genes performed well in 96% of the analyses. Pyrosequencing failed only in 22 out of 540 cases, resulting in missing values for seven out of 27 comparisons and five out of nine SNPs. With the exception of three SNPs (ERD7 SNP1, RUS1 SNP1, and RUS1 SNP2) missing values were extremely rare (Table 2). Repeating the pyrosequencing did not lower the proportion of missing values.

### Comparison between Pool-Seq and individual pyrosequencing

**Figure 1:**
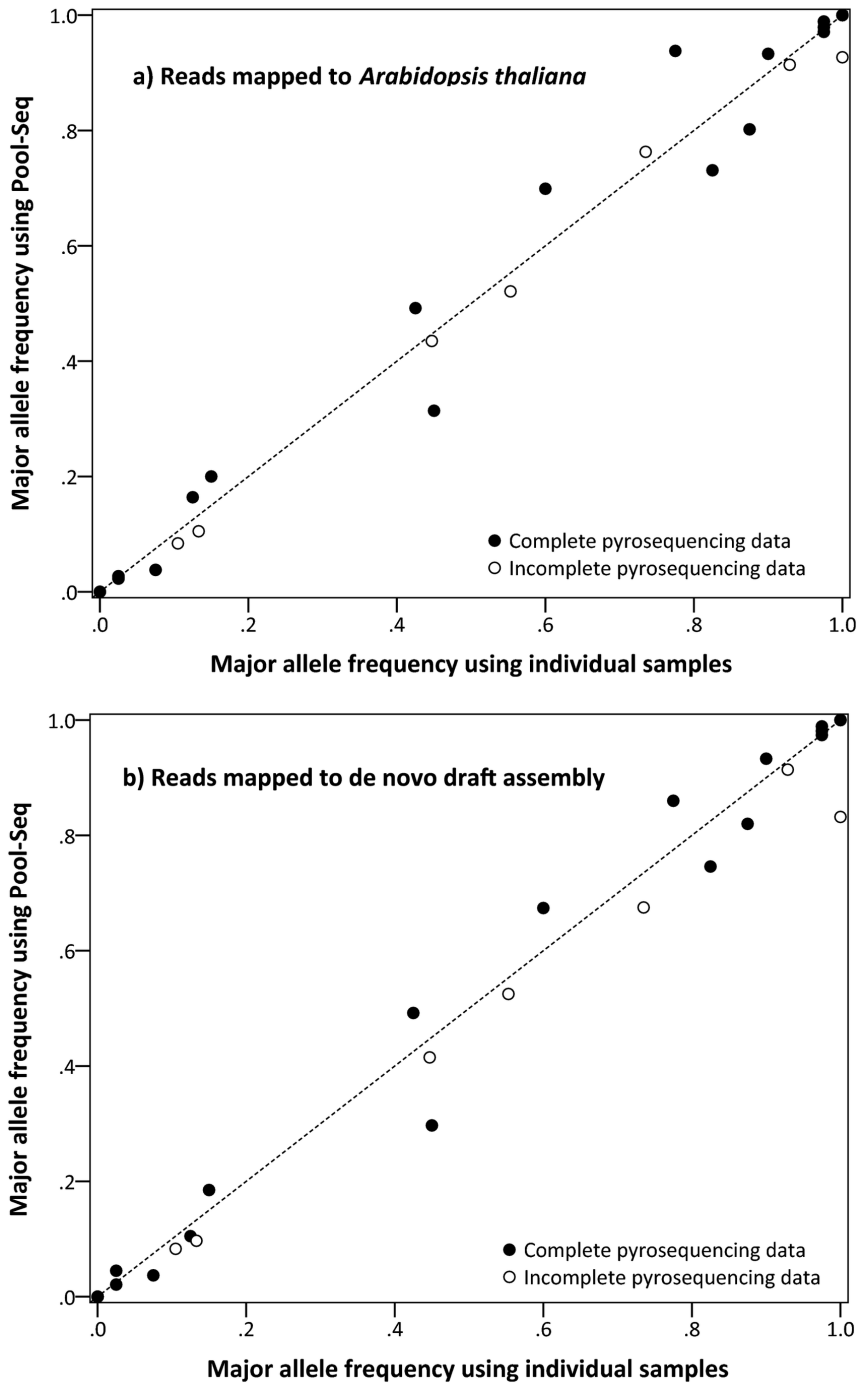
Validation of SNP allele frequencies determined by next-generation sequencing of pooled samples of *Arabidopsis halleri*. Comparison between major allele frequencies calculated from individual genotyping using pyrosequencing (PyroMark) and based on pooled population samples (Pool-Seq) determined with Illumina. In Figure 1a, allele frequencies from the Pool-Seq were calculated from reads mapped to the publicly available *Arabidopsis thaliana* genome. In Figure 1b, the reads were mapped to our own *de* *novo* draft genome of *A. halleri*. Shown are the results of SNPs from six genes for all three populations studied. The dashed line represents the expected 1:1 proportion. Open circles represent comparisons including incomplete date from the pyrosequencing, filled circles refer to comparisons with complete data. Note that some data points are overlapping.

The validation of the estimates of allele frequency from pooled samples mapped against the *A. thaliana* reference genome by individual pyrosequencing was successful (Figure 1a). The average difference between population allele frequencies derived from pooled or individual samples was 3.8% ± 0.8 SE. Only in two out of 27 comparisons, the difference between the allele frequencies was more than 10%, but never exceeded 16.3%. The regression (n = 27, y = 0.996x + 0.001) had an *R* ^2^ of 0.979 and the *p*-value using transformed frequencies was below 0.001. There was no tendency that either the Pool-Seq- or individual-based approach resulted in higher or lower estimates of allele frequency, as the paired *t*-test revealed no difference among the methods (n = 27, *p* = 0.710). Excluding comparisons with missing values from the pyrosequencing led to a very similar result using linear regressions (n = 20, y = 1.000x + 0.006, *R* ^2^ = 0.977, *p* < 0.001) and the paired *t*-test (n = 20, *p* = 0.573). The coverage, ranging from 55 to 269x, had no significant effect on the accuracy of the Pool-Seq, expressed as difference between the two methods (n = 27, *r* = 0.184, *p* = 0.357).

The results using the allele frequency estimates based on the *de novo* assembled reads were highly congruent with those described above. On average, allele frequencies differed by 3.9% ± 0.8 SE, with a maximum of 16.8% (Figure 1b). The linear regression (n = 27, y = 0.985x + 0.001) between the two allele frequency estimates had an *R* ^2^ of 0.979 and was highly significant (*p* < 0.001). The paired *t*-test showed no systematic difference between the methods (n = 27, *p* = 0.221). The same was the case for the analyses excluding SNPs with missing values from the pyrosequencing (linear regression n = 27, y = 1.004x + 0.003, *R* ^2^ = 0.984, *p* < 0.001; paired *t*-test: n = 27, *p* = 0.945). Finally, the coverage (69-284x) had no significant impact (*r* = 0.186, *p* = 0.353) on the accuracy of the Pool-Seq.

### Effect of low coverage on the accuracy of the Pool-Seq

**Figure 2:**
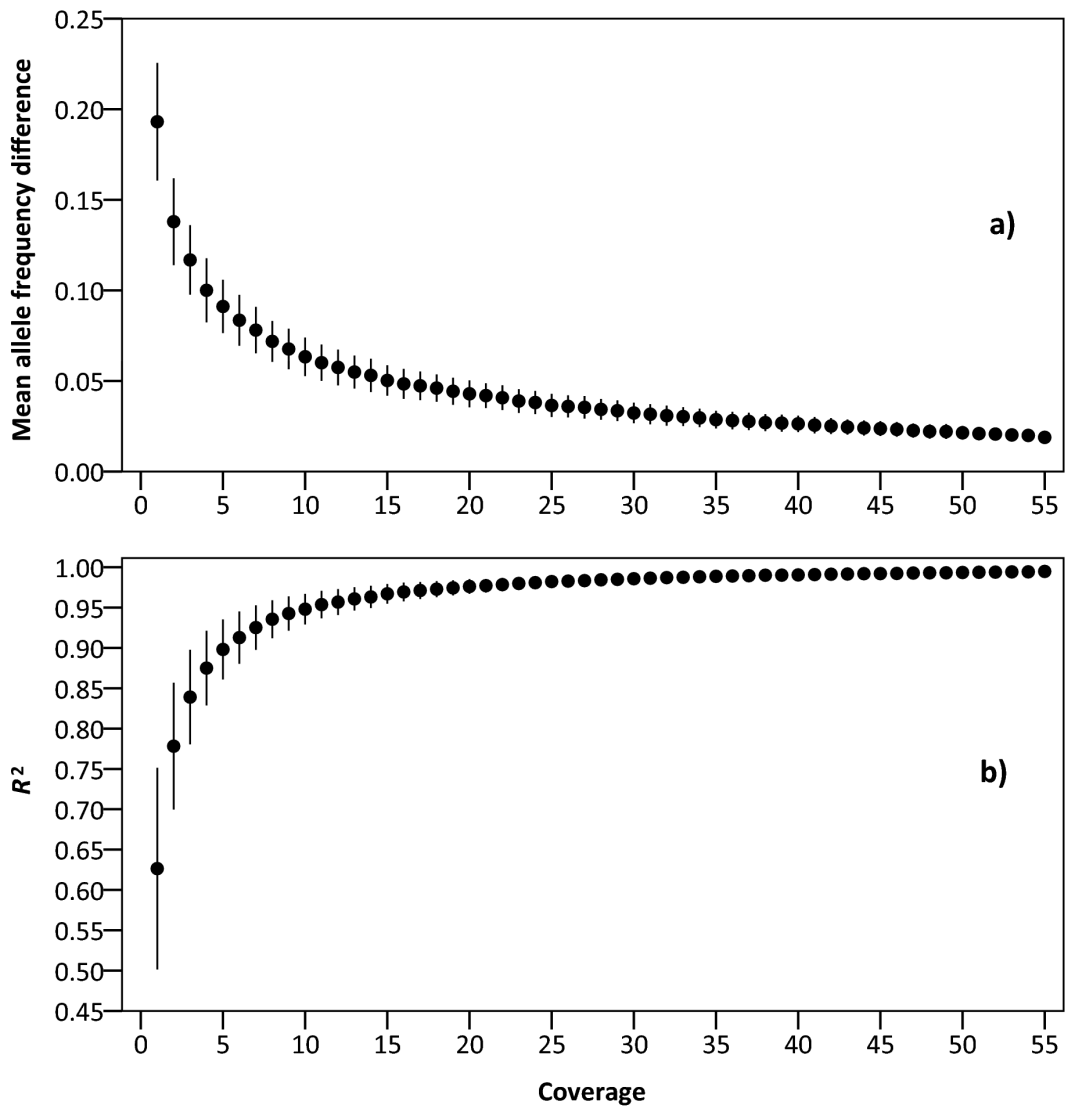
Effect of sequencing coverage on the accuracy of the Pool-Seq in *Arabidopsis halleri*. a) Mean difference between allele frequencies determined by Illumina Pool-Seq and individual pyrosequencing. b) Determination coefficient of the linear regression (*R* ^2^) between the two allele frequency estimates. Data derive from a resampling analysis with 1000 iterations, in which we performed random draws of the reads (mapped to *Arabidopsis thaliana*) in 27 SNP/population combinations. Error bars represent standard deviation.

Our resampling analysis showed that the accuracy of the Pool-Seq increases with increasing coverage (Figure 2). For our data set with 20 diploid individuals per pool, a coverage of at least 16x resulted in a mean frequency difference of <5%, with a coverage of 55x, the mean difference was 1.9% (Figure 2a). To reach an *R* ^2^ of 0.95 in a linear regression, a minimum coverage of 11x was needed, whereas for *R* ^2^ = 0.99 a coverage of 38x was sufficient (Figure 2b).

## Discussion

The use of pooled samples in next-generation sequencing (Pool-Seq) has increased in the last few years, for example to estimate SNP allele frequencies of whole populations (e.g., [4,5,7,8]), because it allows working with large sample sizes while keeping costs and work effort at a minimum [6]. On the other hand, individual haplotype information is lost and the detection of rare alleles may be compromised [14]. Pool-Seq is especially interesting for population genomic studies of local adaptation in natural populations, as they typically require several populations with many individuals to draw reliable conclusions [42]. Regardless of the advantages and disadvantages of the pooled approach, the accuracy of SNP allele frequencies derived from Pool-Seq is the most important criterion to draw accurate population genetic inference. By pyrosequencing specific SNP loci in individual samples, our study is the first to show that Pool-Seq is a highly accurate method for estimating allele frequencies in natural populations.

Our results *on A. halleri* show a good match of the individually determined allele frequencies and the ones estimated by Pool-Seq, irrespective of whether paired-end reads were mapped to a closely related genome (*A. thaliana*) or our own *de novo* draft assembly of *A. halleri* (Figure 1). The slopes of the linear regressions (0.996 and 0.985, respectively) were close to the ideal 1:1 ratio and *R* ^2^-values were high (0.979 for both regressions). Our findings are in concordance with previous studies that have verified Pool-Seq results (Table 1) and mostly found *R* ^2^- or *r*-values above 0.9. However, the studies listed differed in at least one fundamental aspect on how SNP allele frequencies were determined. The estimated allele frequencies from Pool-Seq data were not affected by sequencing coverage above a coverage of 55x. This high range of coverage is a result of the high coverage threshold used in this study, which, in our opinion, should be at least equal to the effective pool size (number of individuals multiplied by the ploidy level) in order to cover rare variants. However, the resampling analysis to evaluate the effect of low coverage shows that, in our case, the Pool-Seq is already quite accurate at much lower coverage (10-20x), at least in terms of regression parameters and allele frequency differences (Figure 2). 

Missing values in the pyrosequencing were present, but did not affect the results, as the regression parameters were similar with or without them. This suggests that no substantial differences between the two measures of allele frequencies exist. Hence, the fact that missing values (i.e. amplification and/or pyrosequencing failures) exist, is unlikely to result from the different platforms used. It rather points to general problems with the template DNA or mapping procedure. As we checked the quality of each DNA sample before pooling, the former is rather unlikely. Therefore, one possibility is that some individuals were so polymorphic in the specific region that (i) their reads did not map to the right position in the mapping process (thereby influencing the development of the pyrosequencing assays), and (ii) PCR and sequencing primers could not bind to the template DNA.

Analyses of sequence data can be strongly facilitated by the availability of a high-quality reference genome of a close relative, like *A. thaliana* [36] used in this study. *Arabidopsis thaliana* and *A. halleri* are estimated to have diverged approximately 5 to 18 million years ago, have different chromosome numbers, and the *A. halleri* genome is about twice the size of the *A. thaliana* genome [43]. Nevertheless, the sequence divergence in coding sequences between the two species was sufficiently low to align 18.1% of the *A. halleri* reads to the *A. thaliana* genome, which resulted in highly accurate allele frequency estimates for the SNPs of interest. Both mapping procedures represent interesting approaches for Pool-Seq studies using non-model organisms. While mapping to a closely related species is a quick and straightforward method, assembling an own draft genome leads to a higher proportion of mapped reads that can be used for further analyses. 

It was beyond the scope of this study to test the effect of pool size on the accuracy of allele frequency estimates of the populations. However, it is clear that increasing the number of individual samples in a pool will decrease the contribution of pipetting and DNA content measurements to the overall error rate [6]. On the other hand, if the detection rate of alleles is an important factor, e.g. if singletons need to be identified, the pool size should be carefully chosen in relation to sequencing error rate of the applied technology [15]. In our case, the error rate of the Illumina data should be below 1% (conservative estimate based on Phred score), therefore the frequency of sequencing errors is well below the frequency of a singleton, which is 2.5% in a pool of 20 diploid individuals. Thus, we can be fairly positive that rare alleles can be detected. Our data confirm this, for example when looking at the results using the mapping against *A. thaliana*: in the three out of 27 comparisons where the minor allele per population represents a singleton, the allele frequency determined by Pool-Seq differed max. 0.4% from the allele frequency determined by pyrosequencing. Moreover, in five out of six comparisons in which loci were inferred to be monomorphic within a population based on pyrosequencing, this was confirmed by Pool-Seq. These results strongly support the accuracy of the pooling approach, as long as pipetting and DNA quantification errors can be held at a minimum, and the coverage is sufficient in comparison to the pool size and to the threshold of read number needed to accept an allele during SNP calling.

As a consequence of its quantitative nature, the pyrosequencing method used for the validation of the Pool-Seq can also itself be applied to accurately determine allele frequencies in pooled samples [28,29]. Therefore, the technology represents an interesting option for studies that need to reliably validate a Pool-Seq protocol by screening the allele frequencies of a limited number of SNPs in pooled samples, because the specific regions are amplified prior to pyrosequencing by using specific primers. Moreover, it could be used for extended studies after having identified SNPs or genes of interest, for example by genotyping additional populations at specific loci to validate results with an independent dataset. However, for screening a large number of individual samples and SNPs, pyrosequencing remains a rather expensive method even with pooled samples.

In conclusion, our validation study shows that allele frequency estimates derived from Pool-Seq can be highly reliable and accurate. The sampling design we used in this study is a typical example for studies investigating population genomics or local adaptation of natural populations [44]. We thus hope that our work will stimulate further studies of natural populations that use a full genome re-sequencing approach to identify genetic variation and evidence for selection in a wide diversity of non-model organisms.

## Supporting Information

File S1 **Mapped consensus sequences of *A. halleri* (mapping approach using the *A. thaliana* genome).** This zip file contains the mapped consensus sequences of the six genes containing the nine validated SNPs, whose positions are given in Table 2. These consensus sequences were compiled from the population specific sequences (only major alleles from each population) and were used for designing the SNP arrays.
(ZIP)

File S2 **Contigs of the *A. halleri* de novo assembly containing the validated SNPs.** This zip file contains the contigs covering the nine validated SNPs, whose positions are given in Table 2. The contigs were assembled using the Brusio population.
(ZIP)
